# Virtuality VS Reality: Testing a virtual approach for the exploratory analysis of Bronze Age urns from Northern Italy

**DOI:** 10.1371/journal.pone.0358226

**Published:** 2026-09-22

**Authors:** Lucia Martina Scalise, Maria Pia Morigi, Claudio Cavazzuti, Rosa Brancaccio, Enrico R. Crema, Marco Seracini, Stefano Benazzi, Emma Pomeroy

**Affiliations:** 1 Department of Archaeology, University of Cambridge, Cambridge, United Kingdom; 2 Department of Physics and Astronomy “Augusto Righi”, University of Bologna, Bologna, Italy; 3 National Institute of Nuclear Physics (INFN) Bologna Division, Bologna, Italy; 4 Department of History and Cultures, University of Bologna, Bologna, Italy; 5 National Institute of Nuclear Physics (INFN), Ferrara Division, Ferrara, Italy; 6 Department of Physics and Earth Sciences, University of Ferrara, Ferrara, Italy; 7 Department of Cultural Heritage, University of Bologna, Ravenna, Italy; 8 Department of Archaeology, Samarkand State University named after Sharof Rashidov, Samarkand, Uzbekistan; Universidad de Sevilla, SPAIN

## Abstract

Cremation is among the most destructive funerary practices. Over the past 30 years, radiographic imaging has increasingly been used to non-destructively extract information from cremation burials. Advances in scanners and imaging software have enhanced the quality of data retrievable through this method. However, scanning cremated remains, particularly when urned, presents challenges due to their fragility, size, material density overlaps, and the high cost of imaging equipment. These factors often result in low-quality images where distinguishing different materials becomes difficult and time-consuming. This exploratory research aims to provide and assess qualitative and quantitative information from tomographic images on burials’ taphonomy, biological profiles (sex and age at death), Minimum Number of Individuals (MNI) and thermal alterations to the bones and to evaluate a relatively simple segmentation strategy to analyse the spatial distribution of the remains in the vessels. For this purpose, 10 non-micro-excavated urns from the Middle-Late Bronze Age necropolis of Vicofertile (Parma, Italy) were scanned using Computerised Tomography (CT), subsequently micro-excavated, and the remains analysed to compare the information obtained from the two approaches. When extracted, data about the age classes, burial taphonomy, and heat-induced changes to the bones (e.g., fractures) were confirmed by the physical analysis, while the MNI, hypothesised to be 10 from the scans, was increased by one. Sex estimation was not possible from the scans. The vertical and horizontal distribution of the remains was estimated for cranial, long, spongy bones, upper, lower limbs, and trunk elements, with the vertical cranial distribution being the most accurately predicted category. Overall, the physical analysis proved essential to refine the results of virtual analysis and deal with the drawbacks of low-quality images. Despite the limitations discussed here, this research presents the largest systematic comparison of tomographic imaging and physical analysis of urned cremations to date, highlighting both the potential and the current limitations of CT-based investigation.

## 1. Introduction

Fragmentation is one of the intrinsic characteristics of burned bones (including cremations). The excavation and subsequent handling of these remains by archaeologists and anthropologists during sieving, washing, and analysis usually further exacerbate this fragmentation [1–3]. Previous studies have used radiographic images to gain as much data as possible from cremations before inevitably causing further fragmentation during the remains’ extraction [4–16]. Since its first use, scholars have acknowledged the usefulness of this inspection in identifying the presence of objects inside the urns, locating the remains, and facilitating their excavation [4]. The technological improvement of the scanners and imaging software has further enriched the quantity and quality of the information retrieved [10].

Scholars have acquired data about the vessels’ preservation, presence of metallic objects, bone fragmentation, bioturbation [6,7,12,13,17–19], and identification of anatomical structures in the assemblage [8,16,20]. Moreover, the volumetric shrinkage of the remains [21], the deposition pattern of the bones, and measurement of their total volume before and after the excavation have been calculated [6,9,14]. Some studies have carried out a more holistic analysis of cremation urns combining virtual and physical approaches. They obtained data regarding the taphonomy of the combustion and burial, grave goods, fragmentation rate of bones, and estimation of the sex and age at death of the deceased [5,10,16,20].

Despite this progress, the virtual analysis of cremated remains has some limitations. The similar densities of the materials and resulting grey values overlap, making their distinction in the images very difficult. Consequently, the segmentation process (i.e., the virtual characterisation of materials based on density values) becomes time-consuming [4,5,8–10,13,16]. Unfortunately, this step is the basis of quantitative analysis (e.g., volumetric and morphometric analyses). To overcome this problem, some researchers have tried MRI (Magnetic Resonance Imaging), which enhances the contrast between bone and other materials embedding it. However, Computerised Tomography (CT) scans seemed more spatially accurate [22]. Gastelum-Strozzi and colleagues [6] tried to develop a semi-automated framework for processing CT data to make it replicable and less time-consuming. Unfortunately, they used a complex approach that would require considerable training. Waltenberger and colleagues [16] used a semi-automatic segmentation averaging the Hounsfield units extracted from different materials. However, a manual correction was often necessary due to the presence of artifacts in the scans, and a few bones could be recognised. Furthermore, the jars’ average size generally prevents using more powerful machines, like µ-CT scanners, that would provide higher resolution images and would simplify the selection of the bones. Alternatives, such as facilities for analysing construction materials [10] or synchrotron facilities [23], exist but are costly and often unavailable locally.

This article presents a novel, simple and relatively quick approach to conducting quantitative and qualitative analysis of urned cremated remains using average-quality tomographic images. The main purpose of this exploratory analysis is extracting information about 1) the urns’ taphonomy, including the impact of factors such as post-depositional disturbance, soil compression, and possible diagenetic alterations 2) the individuals’ sex and age at death, 3) the Minimum Number of Individuals, 4) the thermal alterations to the bones, and 5) the distribution of remains inside the vessels. This last goal would be relevant to identifying potential patterns which might suggest intentional choices and provide insights into funerary behaviours. All these objectives are common to both funerary archaeology and osteological research, regardless of the archaeological context, as they contribute to reconstructing the biological profile of past populations and investigating funerary practices. The present study does not aim to address these archaeological questions in detail. Rather, its objective is methodological: to evaluate a relatively simple approach that maximises the amount of information that can be reliably extracted from average-quality tomographic images to investigate these established research questions. By integrating and validating the virtual procedure through traditional physical analysis, the proposed workflow enabled the retrieval of valuable data on the biological profile and funerary practices of a Late Bronze Age Terramare community (Northern Italy, 1650−1150 BCE). Although validated using this archaeological context, the methodology is applicable to funerary contexts from any period or region. The broader significance of these findings within the archaeological context and their implications for interpreting the community’s life and death will be explored separately. This research primarily focuses on presenting and discussing the analytical approach.

## 2. Materials and methods

### 2.1. Archaeological background and sample selection

**Fig 1:**
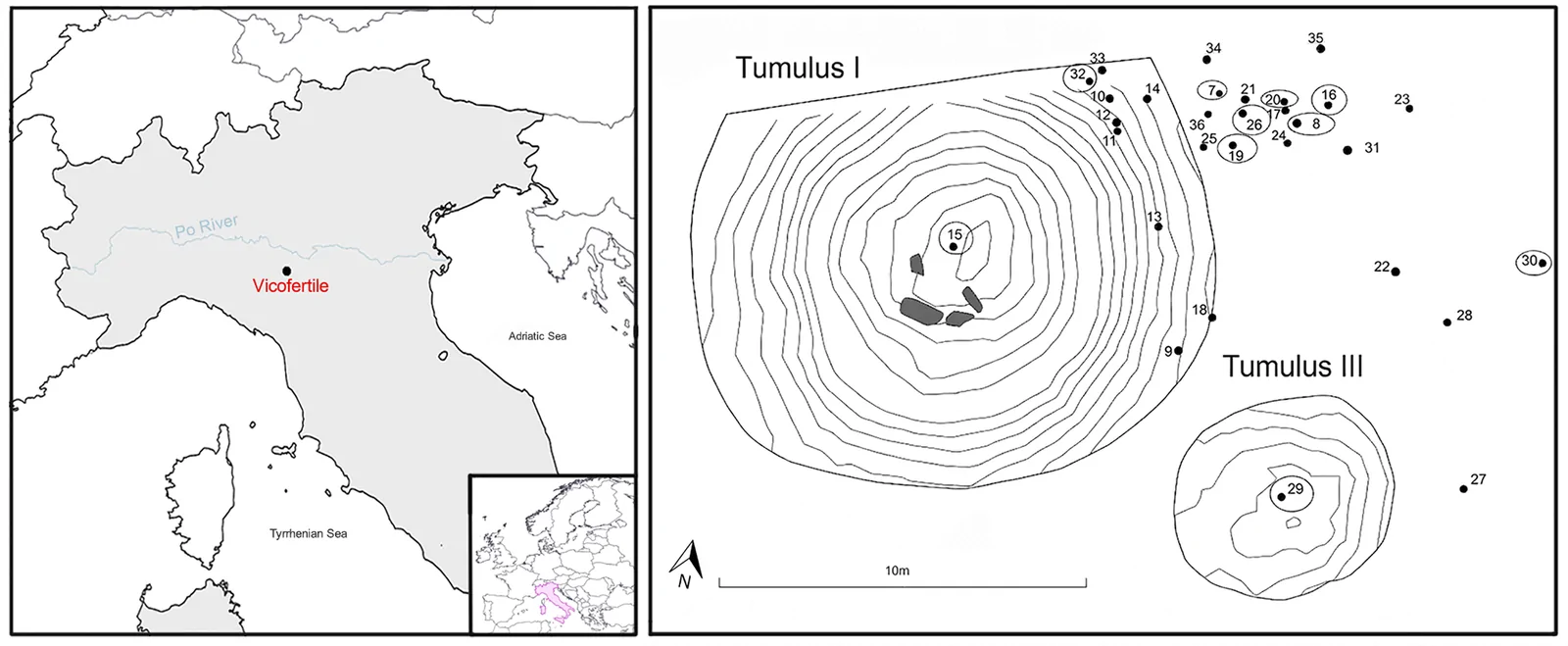
The location of Vicofertile in the proximity of the Po River (Parma, Italy) (left). **Map of Vicofertile (right).** The map of Italy is made with Natural Earth (Free vector and raster map data @ naturalearthdata.com). The numbered black spots on the map of Vicofetile represent the burials. The circled spots show the burials selected for CT scanning. The map of the site is adapted from Ferrari and Mutti [24] and is provided for illustrative purposes only.

A group of 10 urns (S1 Table) was selected from 35 burials excavated in 2009 at Vicofertile, a necropolis discovered during residential development southwest of Parma (Emilia-Romagna, Italy) (Fig 1). All necessary permits were obtained for the described study by the Superintendence of Archaeology, Fine Arts, and Landscape for the provinces of Parma and Piacenza, which complied with all relevant regulations. The remains are currently stored and curated at the Pilotta Monumental Complex in Parma. This research received a favourable ethical opinion from the Department of Archaeology Ethics Board (reference number: ARCH-01-2022-07) at the University of Cambridge (Cambridge, UK). The relative chronology, based on urn and lids shapes and decorations, as well as grave good typology, suggests dating to the advanced Middle Bronze Age (1450/1400–1330/1300 BCE) or the Recent Bronze Age onset (1330/1300–1225/1200 BCE) [24]. As in only a few other Terramare necropolises in the southern Po Plain (e.g., Casinalbo), burial nuclei of varying size were identified at Vicofertile as part of the internal organisation of the cemetery [25]. Two vessels were remarkably buried at the centre of separate tumuli, representing the first evidence of tumulus use in this culture’s necropolises [24] (Fig 1). The Terramare culture spread between 1650 BCE and 1150 BCE across the central Po Plain [26–30], with around 200 settlements and a dozen cemeteries identified across Lombardy, Veneto and Emilia [27,29,30].

The selection of urns for the present study was based on the dimensions and location of the vessels. They showed a good state of preservation to be moved, as they were bounded and excavated *en-bloc*. The vessels buried at the centre of the tumuli (i.e., burial 15 and 29) and the smallest urn (i.e., burial 26 with a maximum circumference of 38 cm) were included. Otherwise, samples from multiple areas of the site were chosen to equally represent the assemblage (Fig 1).

### 2.2. Computerised tomography scans

The number of scans (i.e., 10) was established according to the time available at the X-ray Computed Tomography Laboratory in Ravenna (University of Bologna), where the urns were scanned. The flexible scanning system, designed for cultural heritage objects, includes a cone-beam X-ray source (Smart EVO 200D, 30–200 kV, 6 mA max), an X-ray detector (Hamamatsu C10900D, CsI: TI scintillator, 1216 × 1232 pixel, 100 μm pixel size), and a rotational sample plate. The vessels were scanned with a voltage of 200 kV (the maximum reachable by the X-ray tube), a current of 3.5 mA, and a 1 mm iron plate collimated with a lead mask to reduce X-ray scattering. All scans were completed in two steps (450 projections, 180° angle per step) around 50 minutes each, as the X-ray source could not run continuously, at one-frame average and exposure times of 4.6–5.2 seconds, achieving a final voxel size of 253 μm. Burial 15, with the largest diameter (27 cm) and a prominent position in the largest tumulus, required a modified protocol. The field of view was reduced to concentrate ray power, and the urn was scanned in four steps (two for the upper and two for the lower half). Sections were merged during post-processing using overlapping reference points. This effective but time-intensive method took approximately five hours, making it unsuitable for all scans.

### 2.3. Images post-processing, inspection and segmentation strategy

The reconstruction and correction of tomographic images were carried out using the software PARREC [31], implementing the Feldkamp, Davis, and Kress (FDK) cone-beam algorithm [32]. The post-processing of the images removed artefacts (e.g., outliers, rings caused by X-ray interactions) and the Beam Hardening Correction (BHC) was applied to address selective photon attenuation [33]. The image stacks were adjusted (i.e., alteration of histogram values, brightness, contrast) and filtered with ImageJ’s unsharp mask [34]. The adjusted stacks were imported in Avizo 2022.2 (Thermo Fisher Scientific) for visual examination along XY, XZ, YZ axes. This included assessing the presence of lids, grave goods, urn preservation, compression of remains, and bone fractures (e.g., mosaic, longitudinal, bulls-eye) [35–37]. Observations relevant to estimating sex, age-at-death (methods in 2.4), and anatomical elements were recorded.

**Fig 2:**
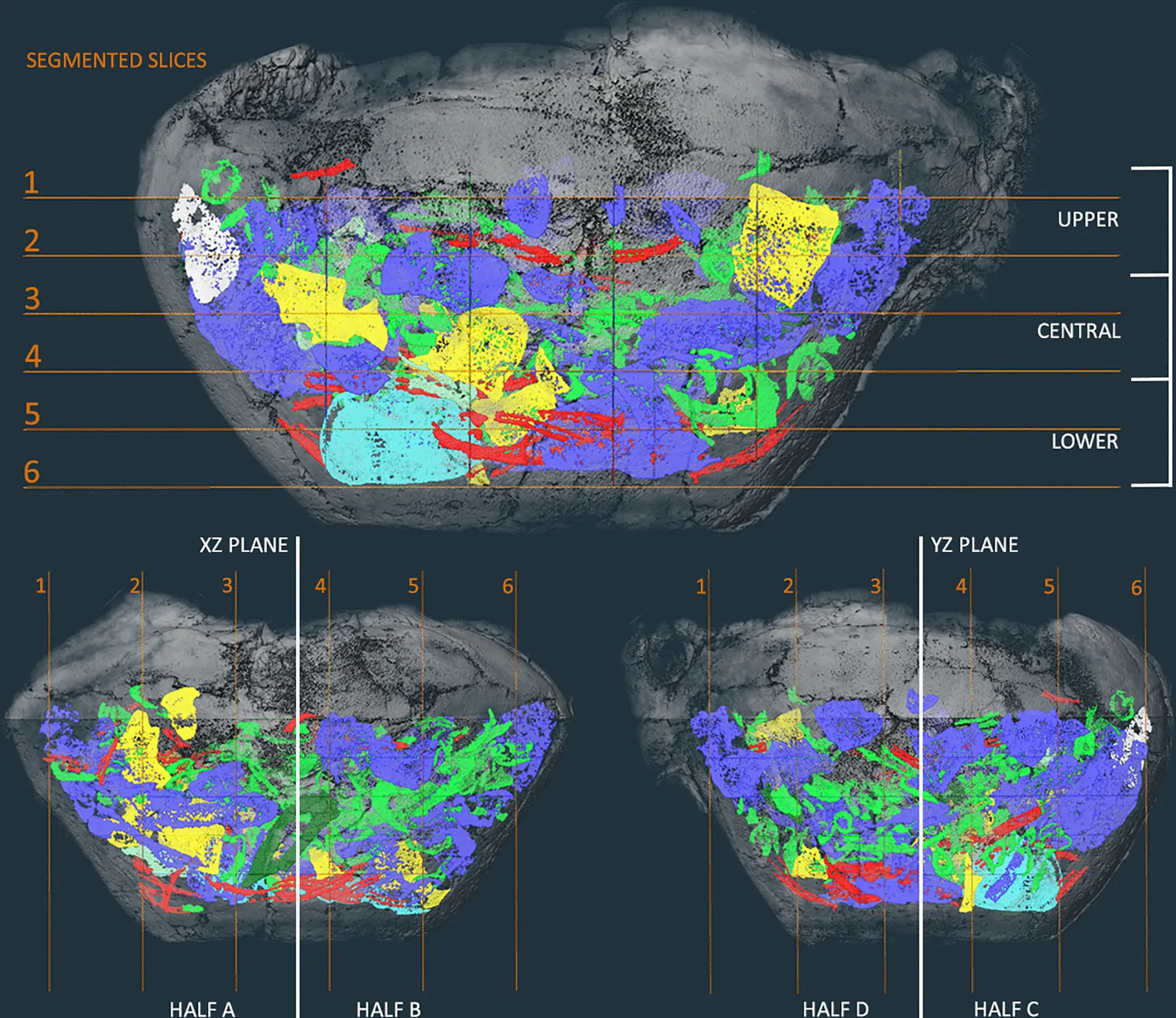
Schematic drawing of slices selection for segmentation and definition of the areas of the vessel. Upper, central, and lower are the vertical sections, while Half A, B, C, and D represent the horizontal sections.

A segmentation strategy was developed to register and quantify the distribution of the remains inside the vessel. Due to the poor contrast between soil, bone, and ceramics, automatic segmentation was unfeasible. The manual segmentation of each slice would have been extremely time-consuming, so six slices for each view were selected and then semi-automatically or manually segmented. Therefore, 18 slices per urn were processed. This sampling was based on the identification of the general range of slices containing recognisable remains from the top to the bottom of the urn. Then, five equal intervals of slices were calculated, and the six slices delimiting the intervals were segmented (Fig 2). This procedure was repeated for each view (XY-XZ-YZ). Depending on the quantity of visible remains, the segmentation took 3–5 days per urn. The number of segmented slices was selected according to the urn’s diameter and the height of the bone deposit, with the intent of keeping the interval between slices at roughly 3.5–4 cm. This spacing was chosen to minimise the risk of overlooking diagnostically relevant elements, which typically measure more than 2–3 cm. Smaller components, such as teeth or phalanges, may not always be captured within a single slice, but they should remain identifiable through qualitative assessment of the CT images. The total number of slices can be modified if a more detailed spatial distribution of elements is needed or in cases where larger urn dimensions require increased sampling resolution.

**Fig 3:**
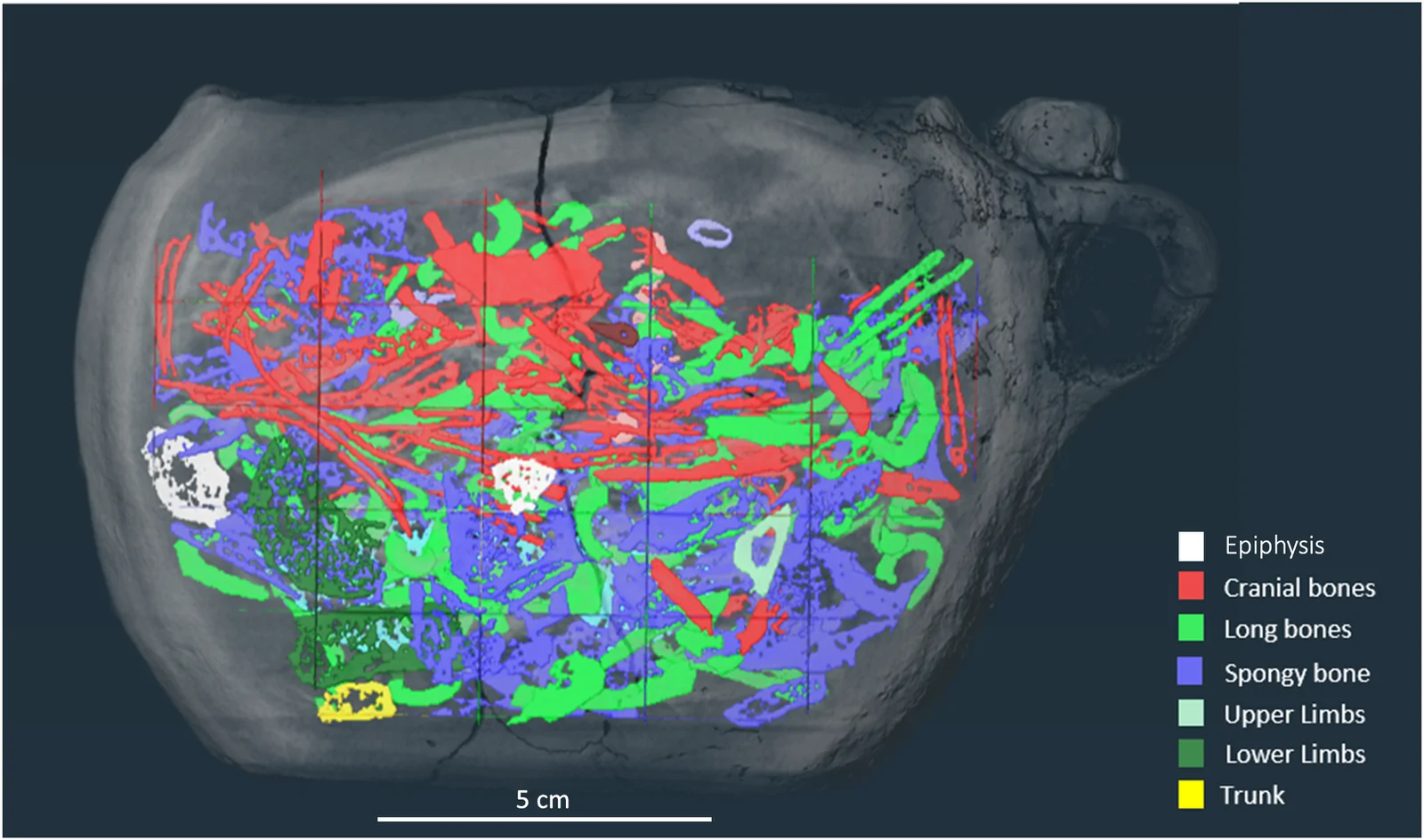
Example of different anatomical categories segmented (burial 26).

Specific anatomical elements were individually labelled when they could be clearly recognised (e.g., tooth, femoral epiphysis). All other segmented bones were grouped into broader categories termed ‘cranial’, ‘spongy’, and ‘long bone’. For the purposes of statistical analysis, three additional classes, ‘trunk’, ‘upper limbs’, and ‘lower limbs’, were introduced, and identified elements were assigned to the most appropriate of these groups (e.g., a femoral epiphysis was placed within the ‘lower limbs’ class) (Fig 3). Elements segmented as generic ‘epiphyses’ that could not be confidently attributed to either the upper or lower limbs were included within the more general ‘spongy bone’ category. Clavicles and scapulae were placed in the ‘upper limbs’ group, and coxal bones within the ‘lower limbs’ (Table 1). Although this grouping may appear anatomically imprecise, it was considered inappropriate to create separate small categories for structures such as the scapular girdle, pelvic girdle, or unassigned epiphyses. Introducing additional groups would have increased the number of variables and resulted in an uneven dataset, with some categories containing only a few elements and others considerably more, which would have placed unnecessary strain on the statistical analysis. Therefore, bones were assigned either to the most similar category in terms of tissue characteristics (e.g., spongy bone and epiphyses) or to the group representing the closest anatomical association (e.g., coxal bones with the lower limbs as components of the appendicular skeleton).

**Table 1:** Classification of anatomical categories and corresponding skeletal elements used in this study.

Category	Anatomical elements
*Cranial bone*	All skull bones
	Teeth
*Trunk*	Hyoid
	Vertebrae
	Sternum
	Ribs
*Upper limbs*	Shoulder girdle
	Arm and hand bones
*Lower limbs*	Pelvic girdle
	Leg and foot bones
*Spongy bone*	Indeterminate epiphyses
	Spongy bone fragments
*Long bone*	Indeterminate long bone fragments

Following segmentation, statistical measurements describing the spatial distribution of the remains were generated in Avizo 2022 2.2. The area (3D) and volume (3D) occupied by each anatomical class (cranial, spongy, long bones, trunk, upper limbs, and lower limbs) were calculated within the upper, middle, and lower sections of each vessel. Although segmentation is performed on individual slices, the resulting measurements are always volumetric, as each slice has a thickness defined by the voxel size (0.253 mm), and the mesh is produced by triangulating the vertices of these volumetric voxels. To define the vertical sections, the total number of slices containing identifiable remains was divided into three equal parts. For the horizontal analysis, the central XZ and YZ planes were used to divide the distribution into halves (Fig 2). Volume and area values were then recorded to quantify the horizontal distribution of each anatomical category across the vessel.

### 2.4. Physical excavation and analysis

The micro-excavation of the urns was conducted at the Bones Lab and ArcheoLaBio, University of Bologna. The scans were employed as guides during the process. Sagittal images were used to estimate the thickness of soil and bone layers. The measure of the latter was divided by three to calculate the upper, central and lower areas for comparability between the virtual and physical layers. The excavation progressed in these three artificial layers. Each layer was also subdivided in four quarters, to register the horizontal distribution of the remains, comparable to the virtual halves. At the beginning of the micro-excavation, the vessel was rotated so that its position corresponded to the orientation of the virtual model. This alignment ensured that the physical quadrants defined during excavation matched the virtual halves identified in the digital reconstruction. Up to 1 cm variance between virtual and physical layers was considered acceptable, as many random variables could affect the excavation (e.g., soil hardness, concretions, uneven surface due to remains position). Identifying elements in tomographic images and the presence of fractures within them prevented blind excavation of delicate components (e.g., epiphyses, diagnostic samples). Layers, diagnostic elements, samples for amelogenin and ^87^Sr/^86^Sr isotope analyses (i.e., *Pars petrosa*, teeth, enamel flakes, fragments of calcined cortical bone and rib) were photographed. The possibility of predicting the presence of these samples from the tomographic images was crucial for avoiding further fragmentation during excavation. Samples were collected from each urn. Unfortunately, the compact, clay-rich soil, dehydrated over time, and hard concretions complicated the excavation. Water had to be used to moisten the soil, but concretions could not be dissolved. Bones were carefully washed with a soft brush and were not sieved to minimize further fragmentation, as handling in the laboratory is known to increase fragmentation [2,38].

Once excavated, each bone was anatomically identified, counted, and weighed individually or, when appropriate, as part of a group of fragments sharing the same anatomical identification, colour, combustion phase, urn, layer, and quadrant. This approach was chosen to record the data at the highest possible level of detail in preparation for future, more complex analyses of the spatial distribution of the remains within the urns. In general, it is always possible to combine data that have been recorded separately when a lower level of detail is required, whereas it is impossible to separate the weight or count of fragments that were originally recorded as a single group. Therefore, a more detailed dataset provides greater flexibility for subsequent analyses. During the physical analysis of the remains, although no quantitative comparison was performed, it became immediately apparent that the sorting of the remains could be carried out more specifically than in the virtual analysis. Still, the same anatomical categories were attributed for comparability. Bone colour, evaluated following Walker and colleagues [39], was used to define three broad combustion phases [39–41]: brown to black, (up to ~550 °C), dark to light grey (~550–800 °C), very light grey to white (>800 °C). Mixed hues were classified by the dominant colour. Fractures and deformations were also assessed [35–37].

Sex was estimated from the cranium and pelvis using standard morphological methods [42,43] and metric methods for postcranial remains [44]. Sex was attributed only when at least three clearly dimorphic features were recognised. It was never estimated exclusively on metric traits, and metrics with accuracy below 80% were excluded [44]. Age at death estimation relied on epiphysis fusion stages [45], tooth development [46,47], and pubic symphysis [48] and auricular surface morphology [49]. The Minimum Number of Individuals (MNI) was estimated from the most frequently occurring skeletal elements, considering sex, age, and pathological lesions [50].

### 2.5. Statistical analysis

Statistical analyses were performed in R version 4.3.3 [51], using the packages “ggplot2” [52] for plotting and “tidyverse” [53] for data handling. A p-value < 0.05 was considered significant. A permutation test was used to evaluate whether there is any difference between the virtual and physical quantitative data in terms of the relative frequencies of different anatomical elements across the vertical and horizontal sections of the urn. The null hypothesis stated that any observed differences were due to random chance, implying no or low correlation between the virtual and physical proportions of elements across sections. In this case, the virtual distribution would not be predictive of the physical distribution. The permutation test was chosen due to the complexity of the nested data structure (six anatomical categories across three sections per urn) and its proportional nature. Each anatomical element’s percentage in each section was calculated over the total of all elements in the urn. Three separate permutation tests were run: one for the vertical distribution (upper, central, lower) and two for the horizontal distribution (Halves A and B, and Halves C and D) (Fig 2). Pearson’s correlation coefficient was calculated between corresponding columns of the two datasets containing the virtual and physical proportions respectively. To test whether these correlations were statistically significant, a null distribution was generated by randomly shuffling the rows of the virtual dataset 1000 times and recalculating the correlations for each permutation. The resulting distribution of permuted correlations was compared to the observed values to determine whether the observed correlations are extreme, indicating a potentially meaningful relationship. For this purpose, a two-sided p-value was derived using a formula adapted from North and colleagues [54]. The code and database for this analysis are provided in Zenodo (DOI: https://doi.org/10.5281/zenodo.15042290).

## 3. Results

**Fig 4:**
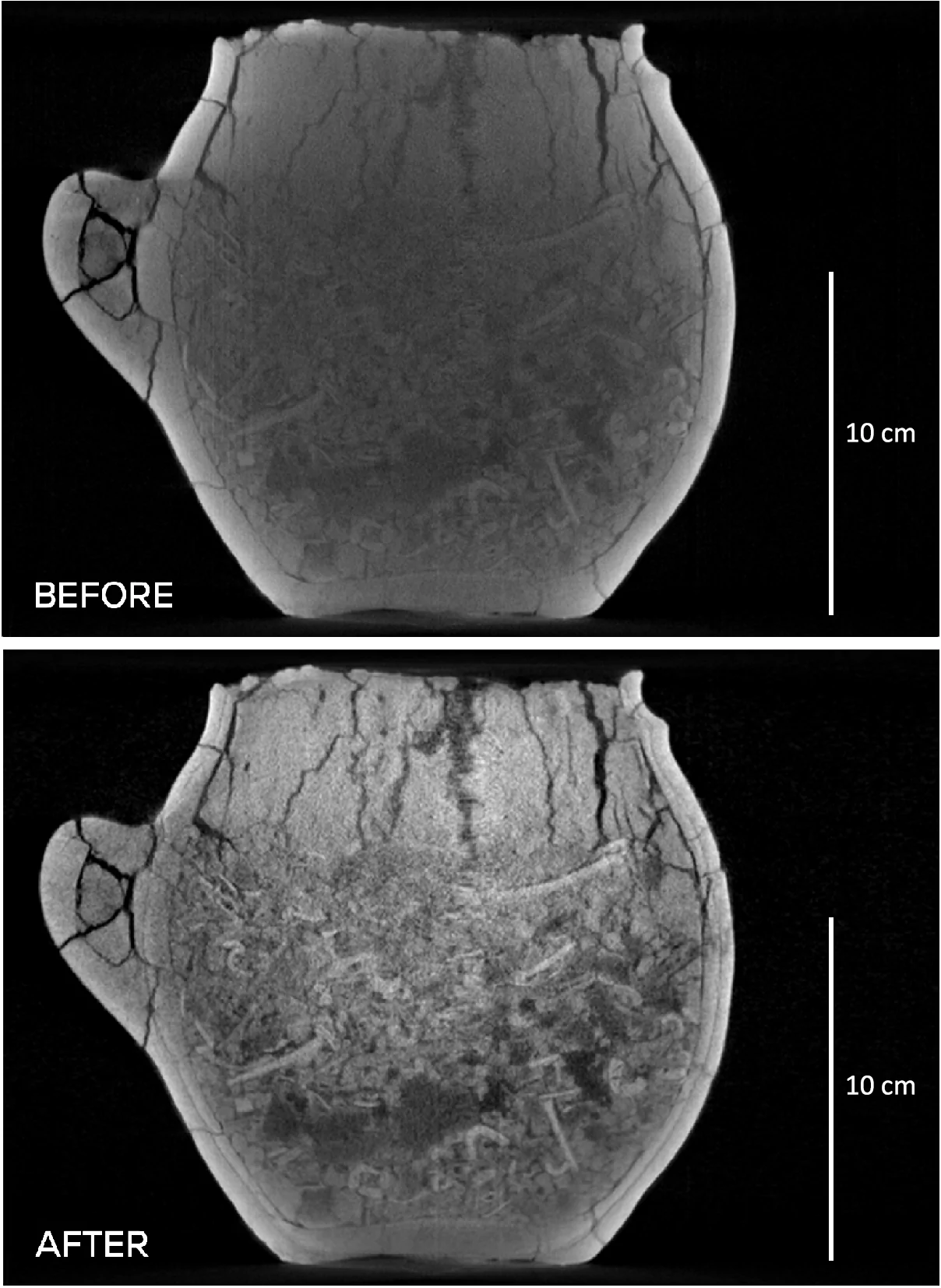
CT images of burial 32 before and after the Beam Hardening correction.

The scanning process presented several challenges. From the initial images, it was evident that the detector signal was low, and the machine’s maximum voltage (200 kV) was insufficient. To compensate, the exposure time was prolonged but capped at 5.2 seconds per frame, as the X-ray tube cannot operate continuously beyond 50 minutes. Despite these adjustments, 8 out of 10 tomographic reconstructions were unsatisfactory due to low resolution and poor contrast, complicating the identification of remains. However, corrections and filtering, particularly the improved BH (Beam Hardening) correction in PARREC, considerably enhanced image quality (Fig 4). Following these processes, only 3 scans (burials 20, 30, 32) remained of poor quality, with burial 29 showing very few discernible remains.

### 3.1. Taphonomy of the burials

The scans were found to be fundamental to assess the state of preservation of the urns and distribution of their contents. In all cases, fractures and cracks in the ceramics were visible and the ‘weakest’ areas identified. In burials 7, 8, 19, and 20, remains appeared clearly compressed towards the bottom of the containers by a thick soil layer. In burial 15, the upper portion of the vessel had collapsed internally, compressing the bone layer. In burial 30 some, large ceramic elements were identified above the remains, nearly vertical, compressing the bones towards the side with the handle. In burial 8, the urn’s bottom was missing, and a rounded metal object (~10 mm) was observed inside the urn.

As expected from the scans, the soil was clay-rich, very dehydrated and hard to excavate. Though soil type was consistent across urns, reddish concretions in burials 15 and 26 made excavation tougher. Despite these drawbacks, virtual and physical layer thickness differed by up to 1 cm in two cases and by ~0.5 cm in others. Burial 29 contained over 800 scattered, thin remains (~2 cm max length), though scans showed few visible remains. In burial 30, scans indicated an 11.5 cm soil layer with pebbles and collapsed ceramics overlying the bones. However, remains appeared after excavating a 2 cm layer, with 369 fragments removed before reaching compressed bones at the bottom. The layer division based on the scan was retained anyway to compare vertical bone distribution between virtual and physical layers.

### 3.2. Biological profiles and thermal alterations to the bones

**Fig 5:**
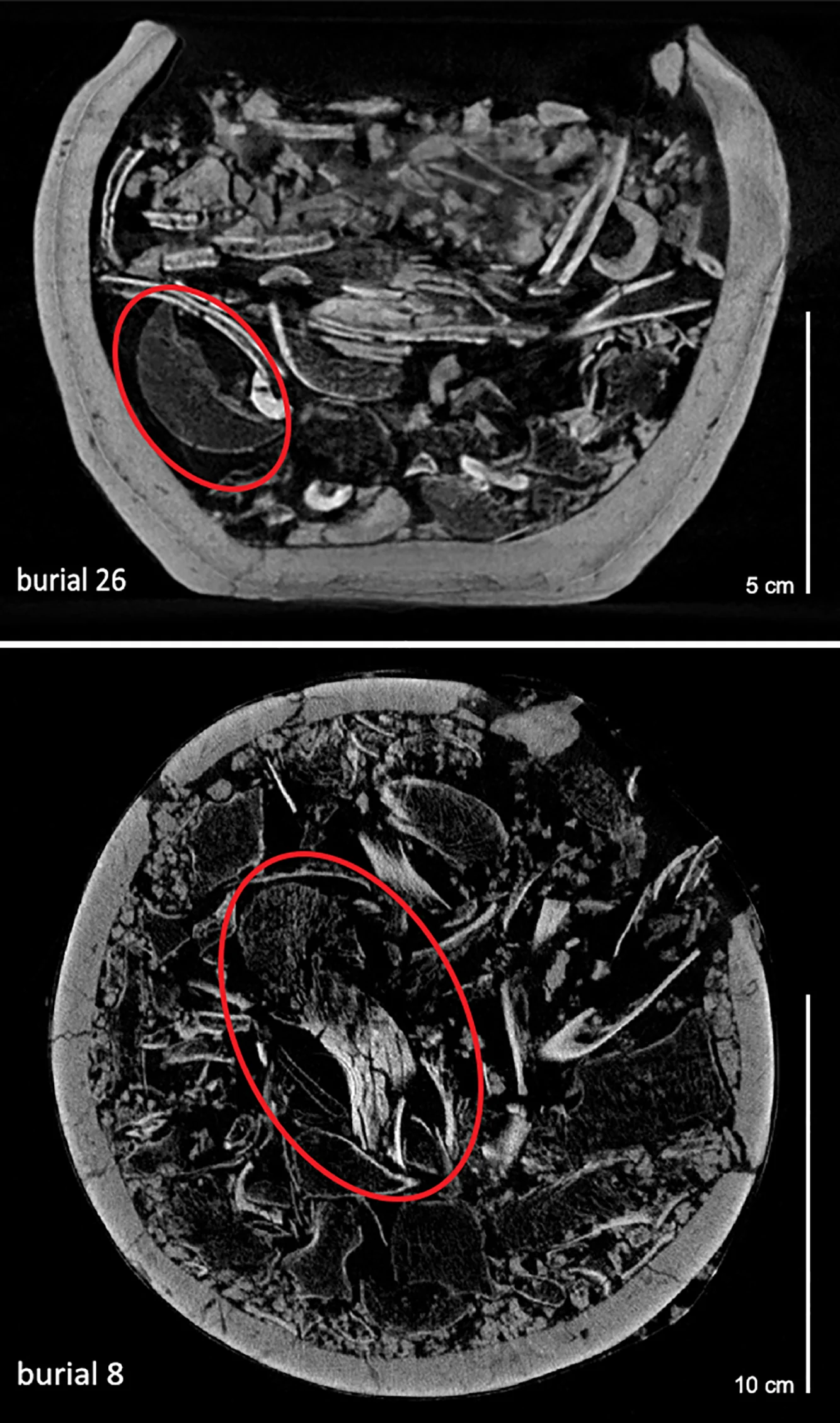
Unfused and fused femoral head from burial 26 and 8, respectively. The unfused element in burial 26 indicates the presence of a non-adult individual, while the fused femur head and neck in burial 8 suggests the presence of an adult individual.

The tomographic images revealed at least one individual per burial. In burial 19, three non-overlapping petrous bone fragments were identified, but the image quality prevented the determination of whether they came from the same individual, so the total MNI based on the tomographic analysis remained 10. Sex could not be assessed due to the lack of dimorphic or measurable elements. Two non-adults (burial 16 and 26) were identified by non-fused humeral and femoral heads (Fig 5). Probable non-adults (burial 19 and 20) were hypothesised based on similar elements, though less distinct. Three adults (burial 7, 8, 15) were identified from fused epiphyses and vertebral end plates (Fig 5). It was not possible to assign to any age class the individuals in burial 29, 30, and 32. Potential samples for chemical analysis (teeth and petrous bones) were identified in all the burials, excluding burial 20 and 29.

**Fig 6:**
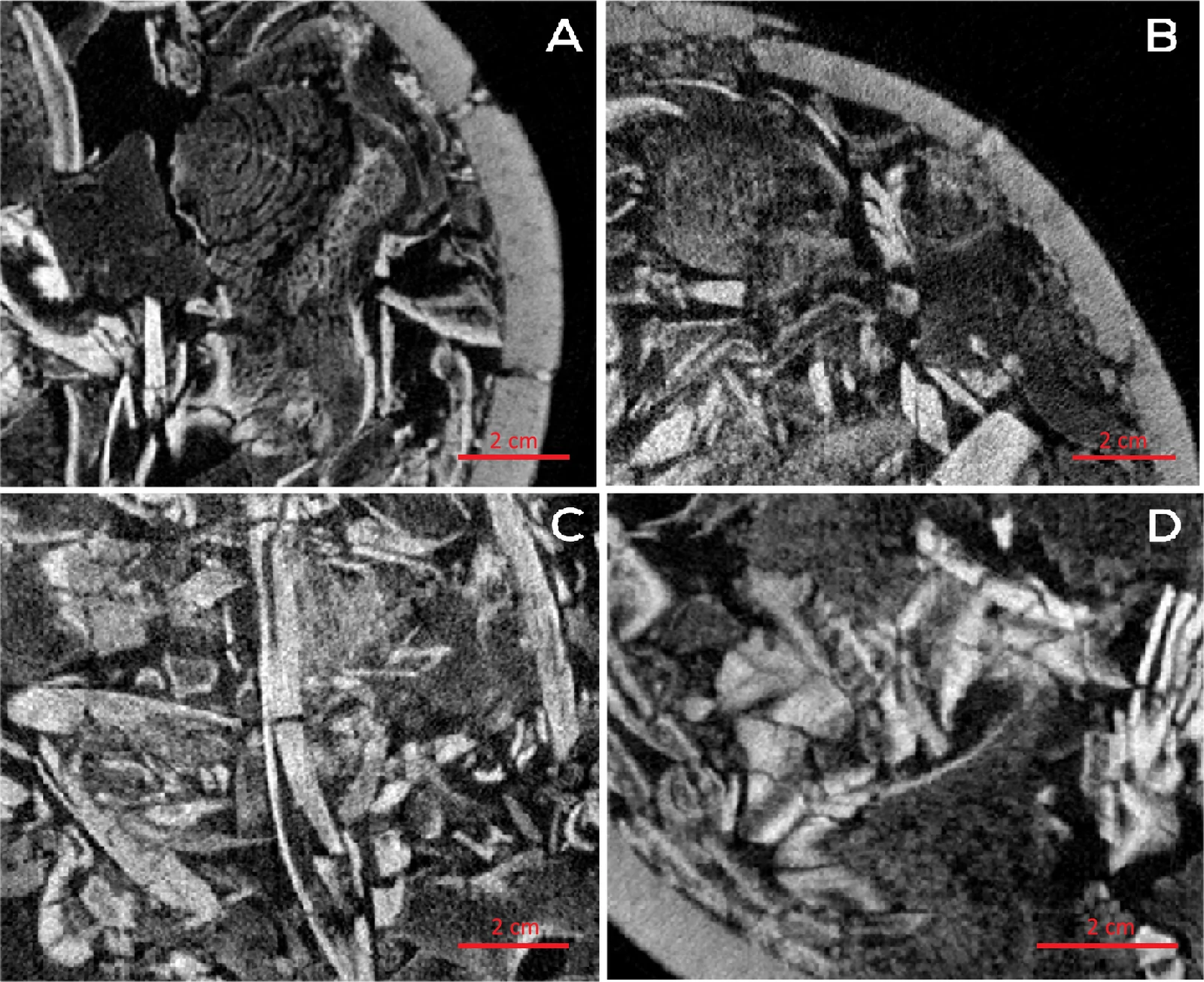
Examples of fracture types identified in burial 15. A: bulls-eye pattern fracture, B: curved fractures, C: transversal and longitudinal fractures, D: mosaic fracture.

Regarding the virtual analysis of the thermal alteration to the bones, only burial 29 completely lacked fractures or deformations. Transversal, longitudinal, and curved fractures occurred in all other burials except burial 30, where curved fractures were absent. Mosaic fractures were visible in five burials, concentric fractures in four, and warping in two (Fig 6).

The physical analysis revealed an MNI of 11, including 6 adults and 5 non-adults. As suggested by the tomographic images, burial 19 contained one adult and one non-adult, identified by age assessment. Two adults were aged 20–40 years, two over 40 years of age, and one over 20. It was not possible to indicate any estimation for the adult individual in burial 19. Non-adults included one aged 3–5, three aged 6–11, and one aged 11–13. One adult was female (Table 2). All potential chemical analysis samples identified in the scans were collected without further damage. Of all the remains, 76.3% showed fractures, deformation, or warping, including burial 29. Around 92.4% are calcined (phase 3), 6.8% are in phase 2, and 0.4% in phase 1, consistent with high burning temperatures of at least 700/800 ºC [40].

**Table 2:** Summary of the results of sex and age-at-death estimation from virtual and physical analysis.

Burial	Age-at-death (Virtual)	Age-at-death (Physical)	Sex	Fractures and deformations (Virtual)	Fractures and deformations (Physical)
**7**	Adult	>40 yrs	ND	transversal, longitudinal, curved	transversal, longitudinal, curved, mosaic
**8**	Adult	>40 yrs	F	transversal, longitudinal, curved, mosaic, concentric, warping	transversal, longitudinal, curved, mosaic, concentric, warping
**15**	Adult	20-40 yrs	ND	transversal, longitudinal, curved, mosaic, concentric, warping	transversal, longitudinal, curved, mosaic, concentric, warping
**16**	Non-adult	11-13 yrs	ND	transversal, longitudinal, curved, mosaic	transversal, longitudinal, curved, mosaic, concentric, warping
**19**	Non-adult?	7-11 yrs	ND	transversal, longitudinal, curved, mosaic	transversal, longitudinal, curved, mosaic, warping
**19**	Not recognised	Adult	ND	transversal, longitudinal, curved, mosaic	transversal, longitudinal, curved, mosaic, warping
**20**	Non-adult?	6-8 yrs	ND	transversal, longitudinal, curved, concentric	transversal, longitudinal, curved, mosaic, concentric, warping
**26**	Non-adult	7-9 yrs	ND	transversal, longitudinal, curved, mosaic, concentric, warping	transversal, longitudinal, curved, mosaic, concentric, warping
**29**	Not determinable	3-5 yrs	ND	Not observable	transversal, longitudinal, curved, mosaic
**30**	Not determinable	20-40 yrs	ND	transversal, longitudinal	transversal, longitudinal, curved, mosaic, warping
**32**	Not determinable	> 20 yrs	ND	transversal, longitudinal, curved	transversal, longitudinal, curved, mosaic, warping

### 3.3. Distribution of the remains inside the vessels

**Fig 7:**
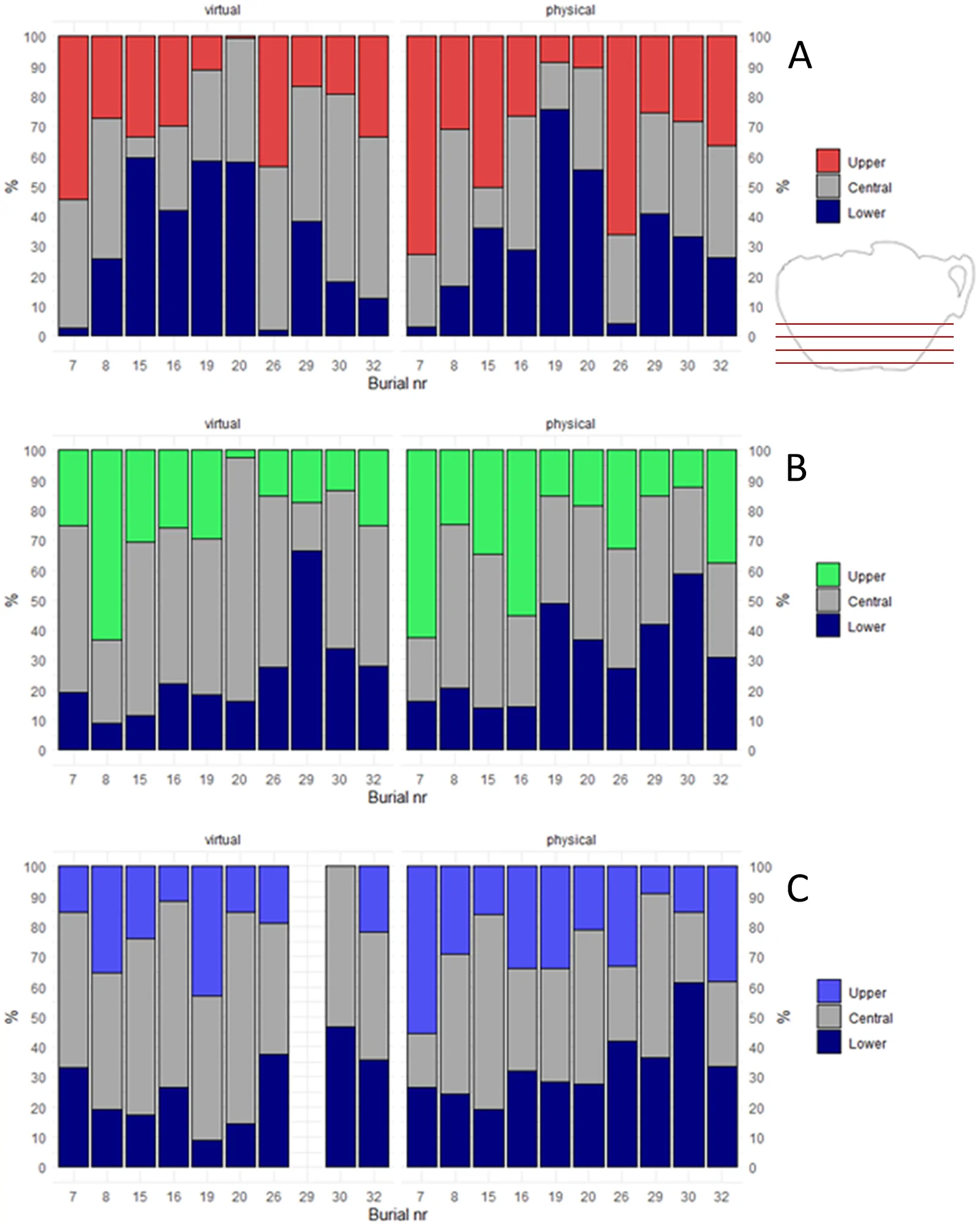
Vertical distribution of remains. Cranial bones (A), long bones (B), and spongy bone (C) in the upper, central, and lower layers of the vessels resulting from the virtual (left) and physical (right) analysis.

The vertical distribution of cranial bones appeared similar between virtual and physical analyses. Virtual data showed cranial remains concentrated in the upper or upper and central layers for burials 7, 8, 26, 30, and in the lower or lower and central portions for burials 15, 16, 19, 20, and 29. The physical observations differed in burials 15 and 30, where cranial remains were found mainly in the upper and lower layer respectively (Fig 7A). In the virtual vertical distribution, long bones were more abundant in the upper, and upper and central sections in five burials (7, 8, 15, 16, 19), and in the lower, and lower and central areas in the others. Physical analysis reversed the trend only in burial 19. In all the other cases, the general trend was confirmed (Fig 7B). Virtual analysis showed spongy bone concentrated in the lower/central areas in five burials (7, 16, 26, 30, 32), with none identified in burial 29. In the physical analysis, however, spongy bone was found in this burial primarily in the central lower area. The correspondence between virtual and physical distribution reversed in five cases. Burials 7, 16, and 32 showed more abundant spongy bone in the upper and upper-central sections, while burials 15 and 20 were the opposite (Fig 7C).

**Fig 8:**
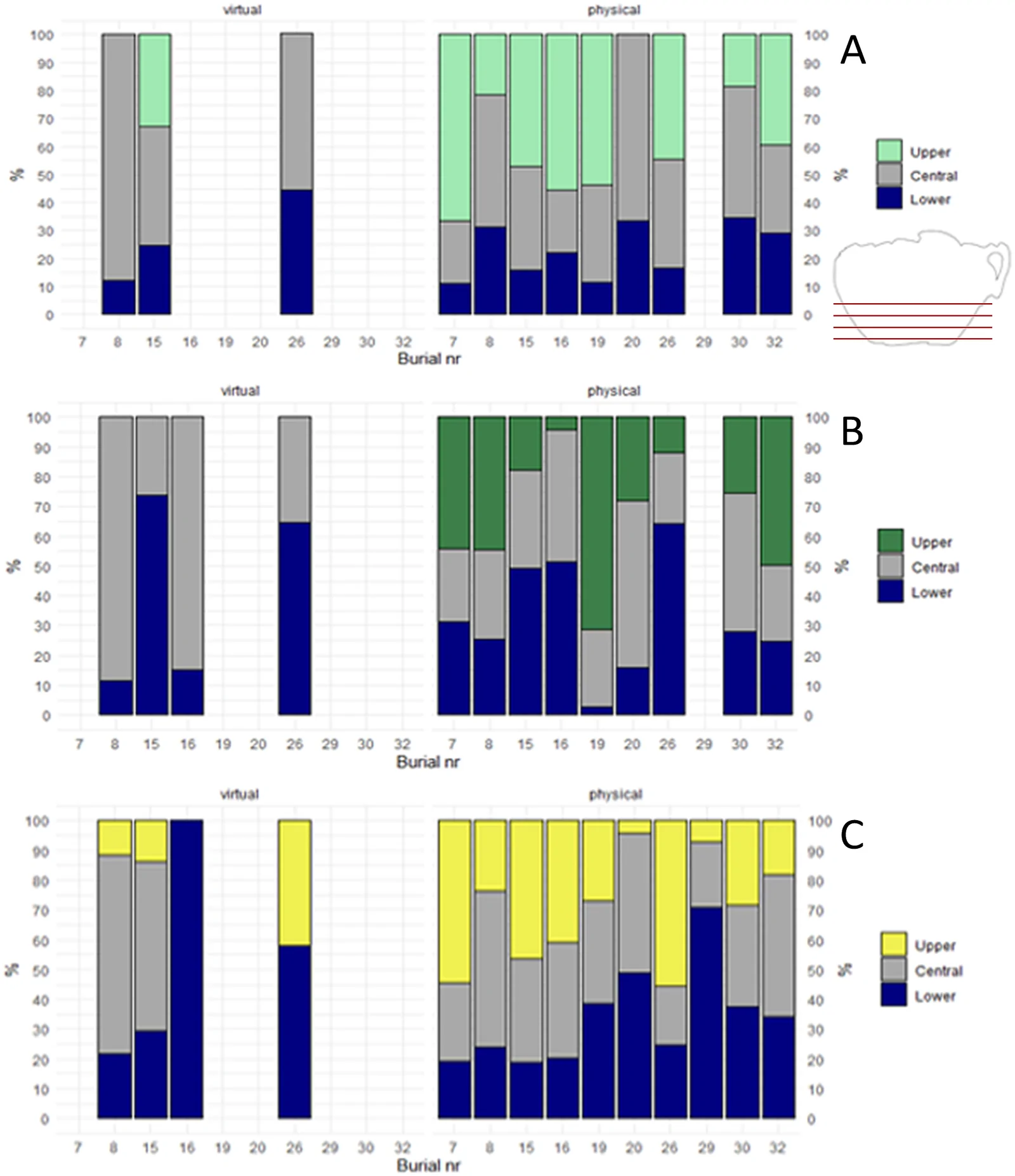
Vertical distribution of remains. Upper limbs (A), lower limbs (B), and trunk remains (C) in the upper, central, and lower layers of the vessels resulting from the virtual (left) and physical (right) analysis.

Upper or lower limb remains were rarely distinguishable in tomographic images. Upper limb elements were recorded in burials 8, 15, and 26 while lower limbs were observed in these burials as well as in burial 16. Most identifiable limb bones were in the central and lower areas of the urn, except in burial 15, where upper limbs were in the upper and central sections. These patterns were confirmed in most cases by the physical analysis, although a small proportion of limbs fragments were identified after the excavation (1132 fragments – 7.3% of total remains). In burial 26, upper limb bones were found in the upper section, while lower limb bones were in the upper section of burial 8. In general, a much more detailed quantification of the remains was made for both categories from the physical analysis, except in burial 29, where no elements of these categories were identified. Upper limbs were more abundant in the upper areas, and lower limbs were more evenly distributed across sections (Fig 8A-8B).Trunk remains were identified in four burials (8, 15, 16, 26) via CT scans, mostly in lower or central areas of the urns. A relatively small number of remains could be identified in this category during the physical analysis (1456 fragments – 9.4%). Trunk remains were found predominantly in the central and lower portions of the urn (Fig 8C).

**Fig 9:**
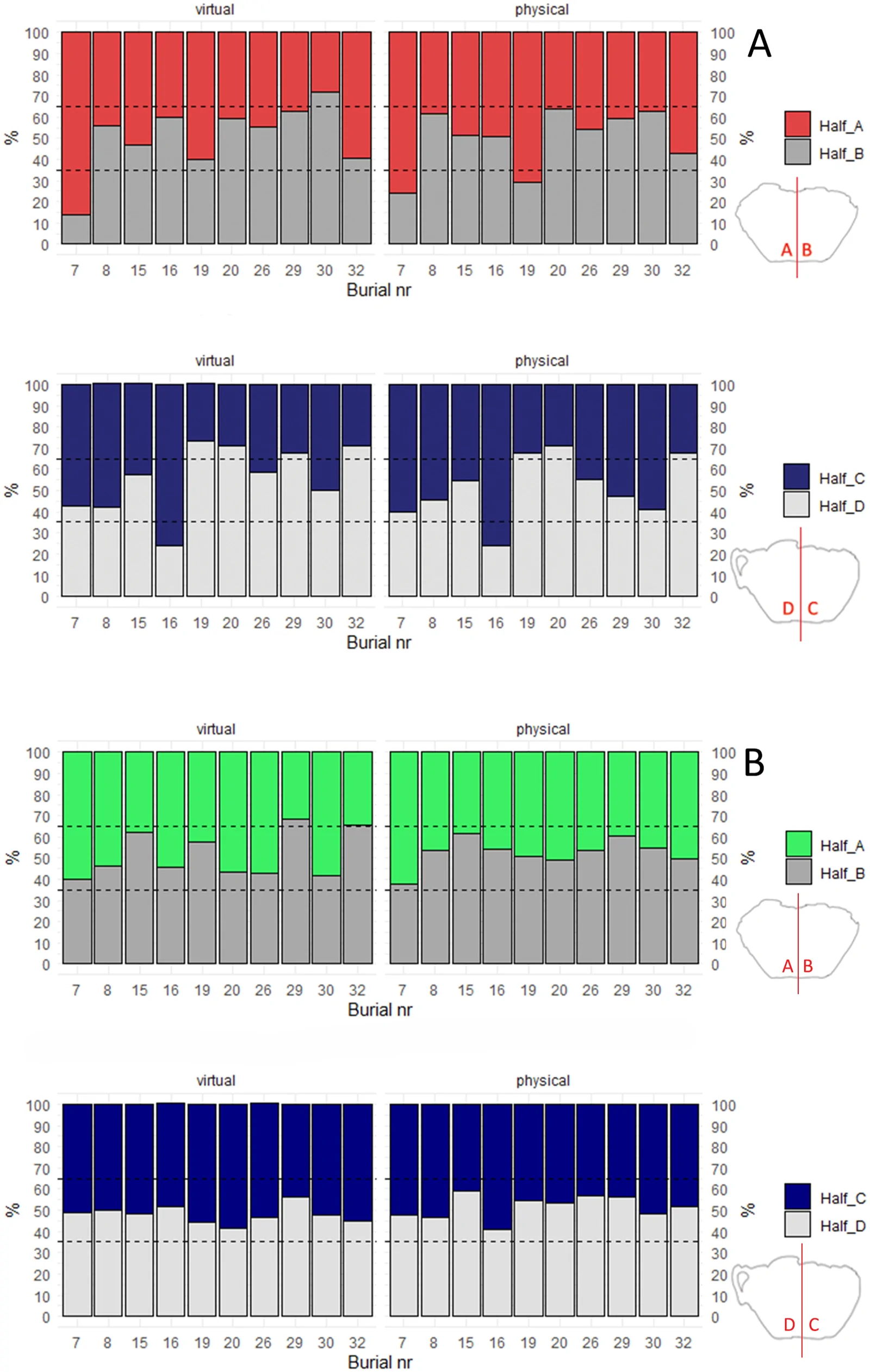
Horizontal distribution of remains. Cranial (A) and long (B) bones in the four halves of the vessels resulting from the virtual (left) and physical (right) analysis. The dashed horizontal lines indicate the threshold used to define a substantial predominance (65%) of an element within one of the two halves.

For the horizontal distribution, we considered a threshold of at least 65% in one of the two halves (A or B – C or D – Fig 2) as substantial because, unlike the vertical distribution, where a central section could be calculated between the upper and lower layers, no equivalent middle layer was available. Most cranial bones were evenly distributed, with some exceptions showing higher abundance in specific halves: burial 7 in half A, burial 16 in half C, and burials 19, 20, and 32 in half D. These trends were confirmed virtually and physically, except for burial 30 (half B) and burial 29 (half D) (Fig 9A). Long bones were similarly uniform, with burials 29 and 32 showing more remains in half B, but this was not confirmed physically (Fig 9B).

**Fig 10:**
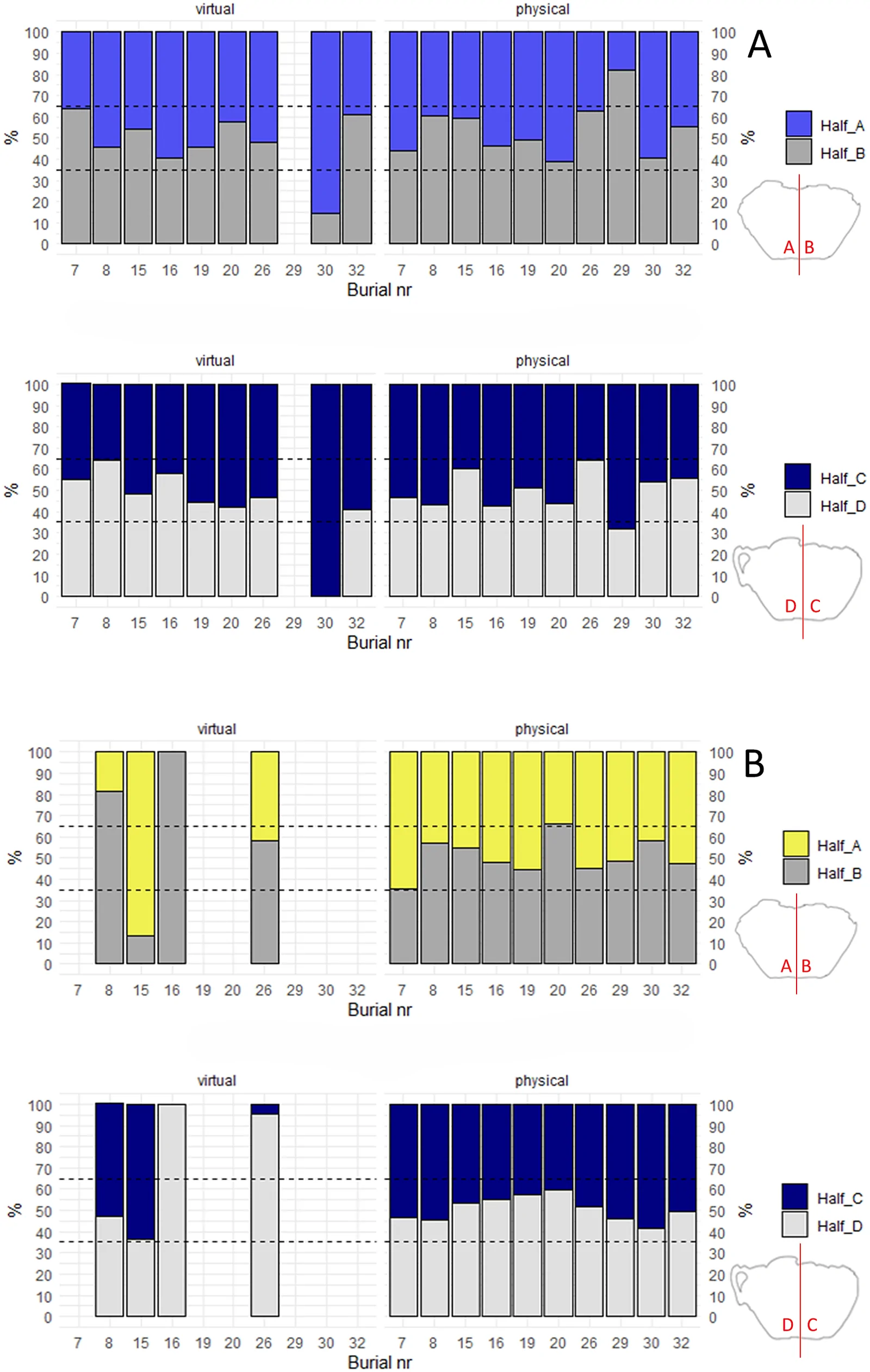
Horizontal distribution of remains. Spongy bone (A) and trunk remains (B) in the four halves of the vessels resulting from the virtual (left) and physical (right) analysis. The dashed horizontal lines indicate the threshold used to define a substantial predominance (65%) of an element within one of the two halves.

**Fig 11:**
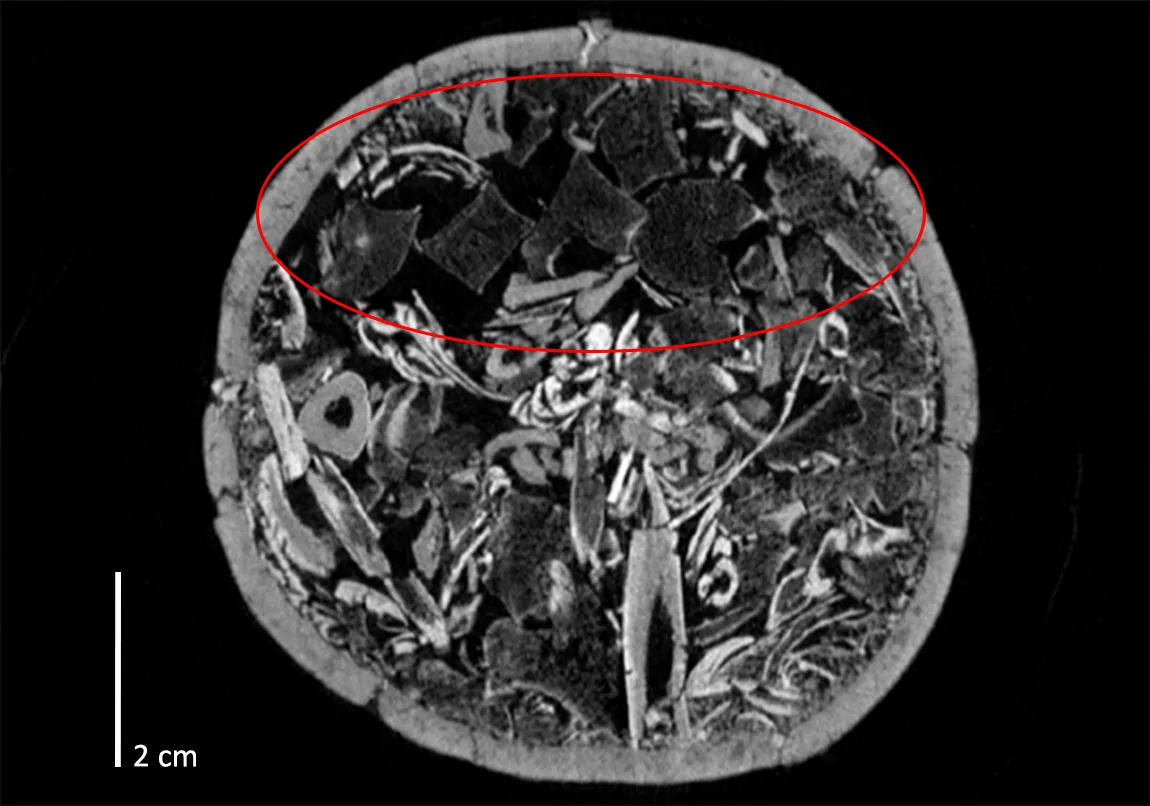
Burial 15: Tomographic image of vertebrae grouped (red circle) on one side (half A) of the urn.

No consistent patterns were observed between virtual and physical distributions for spongy bone. In burial 30, the virtual prevalence in halves A and C was not confirmed physically, while in burial 29, spongy bone was more frequent in halves B and C post-excavation (Fig 10A). No patterns of trunk remains determined virtually were confirmed in the physical results (Fig 10B). In the virtual analysis of burial 15, trunk remains were more abundant in half A as some vertebrae arranged in a row were clearly visible (Fig 11). This group of vertebrae was also retrieved during the excavation. Still, trunk remains that were not identifiable in the scans were found in other areas, making the final physical distribution count more even between the halves.

**Fig 12:**
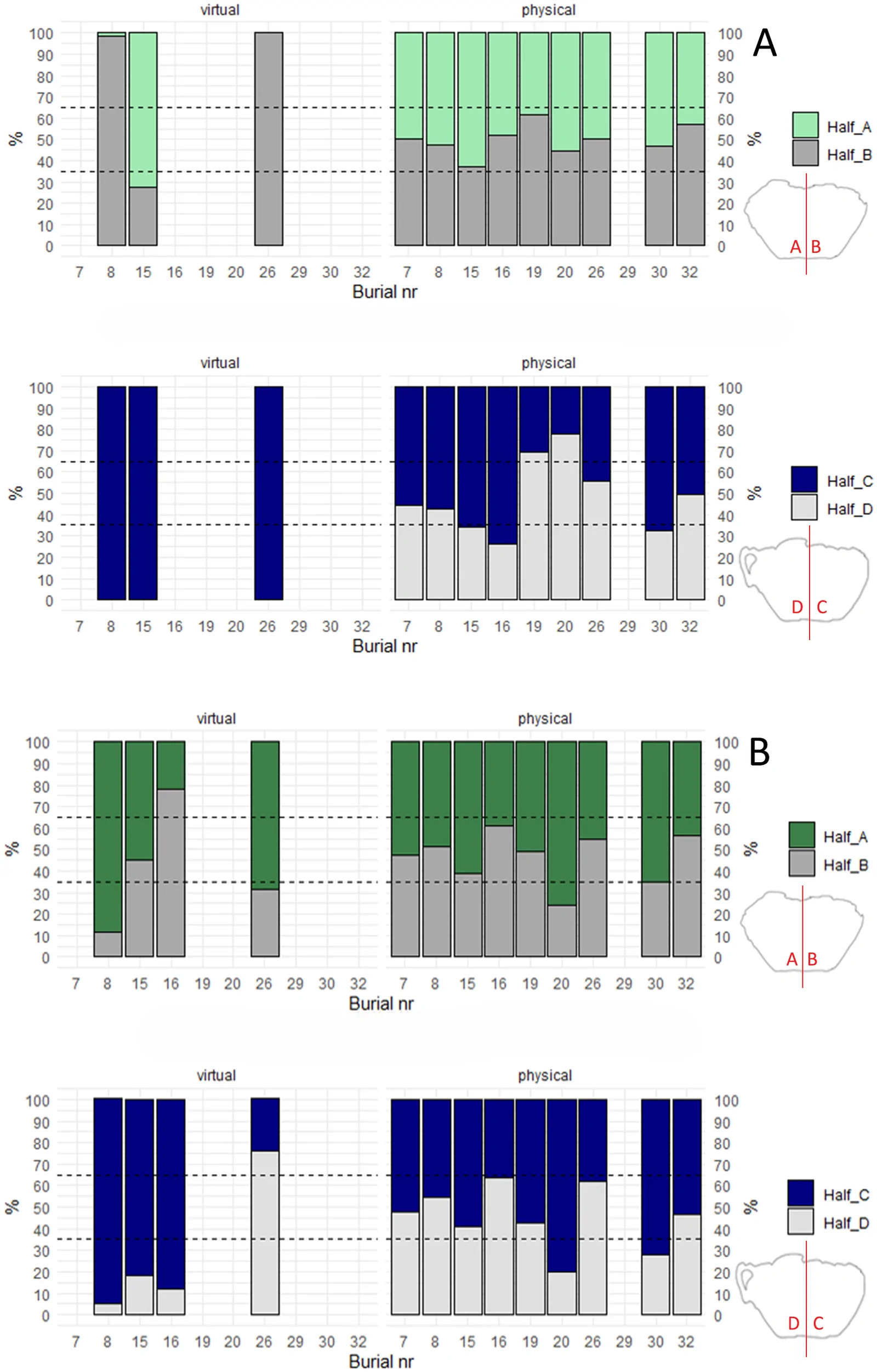
Horizontal distribution of remains. Upper (A) and lower (B) limbs in the four halves of the vessels resulting from the virtual (left) and physical (right) analysis. The dashed horizontal lines indicate the threshold used to define a substantial predominance (65%) of an element within one of the two halves.

High values from the virtual analysis for upper limb elements in burials 8, 15, and 26 were not confirmed physically. However, more upper limb remains were found in half C (burials 15, 16, 30) and half D (burials 19, 20). Similar discrepancies occurred for lower limbs, with more fragments found in halves A and C in burials 20 and 30 (Fig 12A-12B).

The permutation analysis results for vertical distribution (Table 3) show strong correlations (>0.7) between virtual and physical data for cranial bones, with the highest value (0.986) in the lower layer. A correlation of 0.759 was observed for lower limbs in the lower area. Significant p-values were reported for these categories and upper limb remains in the urns’ upper portion, despite a weaker correlation (0.646). For 6 variables out of 18 of the vertical distribution, the null hypothesis was rejected. Weaker correlations were found for ‘Spongy Bone Lower’ (0.646) and ‘Trunk Central’ (0.617), with p-values near significance (ca. 0.05). For the horizontal distribution, strong correlations were observed for cranial remains in halves B (0.915), C (0.830), and D (0.896), and for spongy bone in half D (0.716). Significant p-values supported coefficients >0.6, including trunk remains in half D (0.632, p = 0.020). No elements were recovered for upper limbs in half D. The null hypothesis was rejected for 8 of 24 variables (Table 3).

**Table 3:** Permutation test results for virtual–physical data correlation. Summary of the observed correlation between the virtual and physical data and p-values obtained from the permutation analysis for each variable. Bold indicates significant values.

Variable	Correlation	Two-sided p-value
Cranial Bones Upper	**0.790**	**0.012**
Cranial Bones Central	**0.719**	**0.026**
Cranial Bones Lower	**0.986**	**0.002**
Long Bones Upper	−0.081	0.839
Long Bones Central	−0.211	0.619
Long Bones Lower	0.209	0.593
Spongy Bone Upper	0.406	0.304
Spongy Bone Central	0.589	0.074
Spongy Bone Lower	**0.646**	**0.034**
Upper Limbs Upper	**0.656**	**0.002**
Upper Limbs Central	0.275	0.390
Upper Limbs Lower	0.036	0.645
Lower Limbs Upper	−0.409	0.190
Lower Limbs Central	0.481	0.170
Lower Limbs Lower	**0.759**	**0.032**
Trunk Upper	0.567	0.138
Trunk Central	0.617	0.060
Trunk Lower	−0.257	0.482
Cranial Bones Half A	**0.688**	**0.042**
Cranial Bones Half B	**0.915**	**0.004**
Long Bones Half A	0.028	0.975
Long Bones Half B	−0.203	0.597
Spongy Bone Half A	**0.690**	**0.026**
Spongy Bone Half B	0.580	0.098
Upper Limbs Half A	0.536	0.342
Upper Limbs Half B	0.071	0.827
Lower Limbs Half A	0.341	0.320
Lower Limbs Half B	**0.708**	**0.012**
Trunk Half A	0.278	0.478
Trunk Half B	0.573	0.020
Cranial Bones Half C	**0.830**	**0.006**
Cranial Bones Half D	**0.896**	**0.006**
Long Bones Half C	0.001	0.911
Long Bones Half D	−0.345	0.330
Spongy Bone Half C	0.582	0.066
Spongy Bone Half D	**0.716**	**0.028**
Upper Limbs Half C	0.378	0.286
Upper Limbs Half D	NA	NA
Lower Limbs Half C	0.464	0.204
Lower Limbs Half D	0.367	0.250
Trunk Half C	0.459	0.146
Trunk Half D	**0.632**	**0.020**

**Fig 13:**
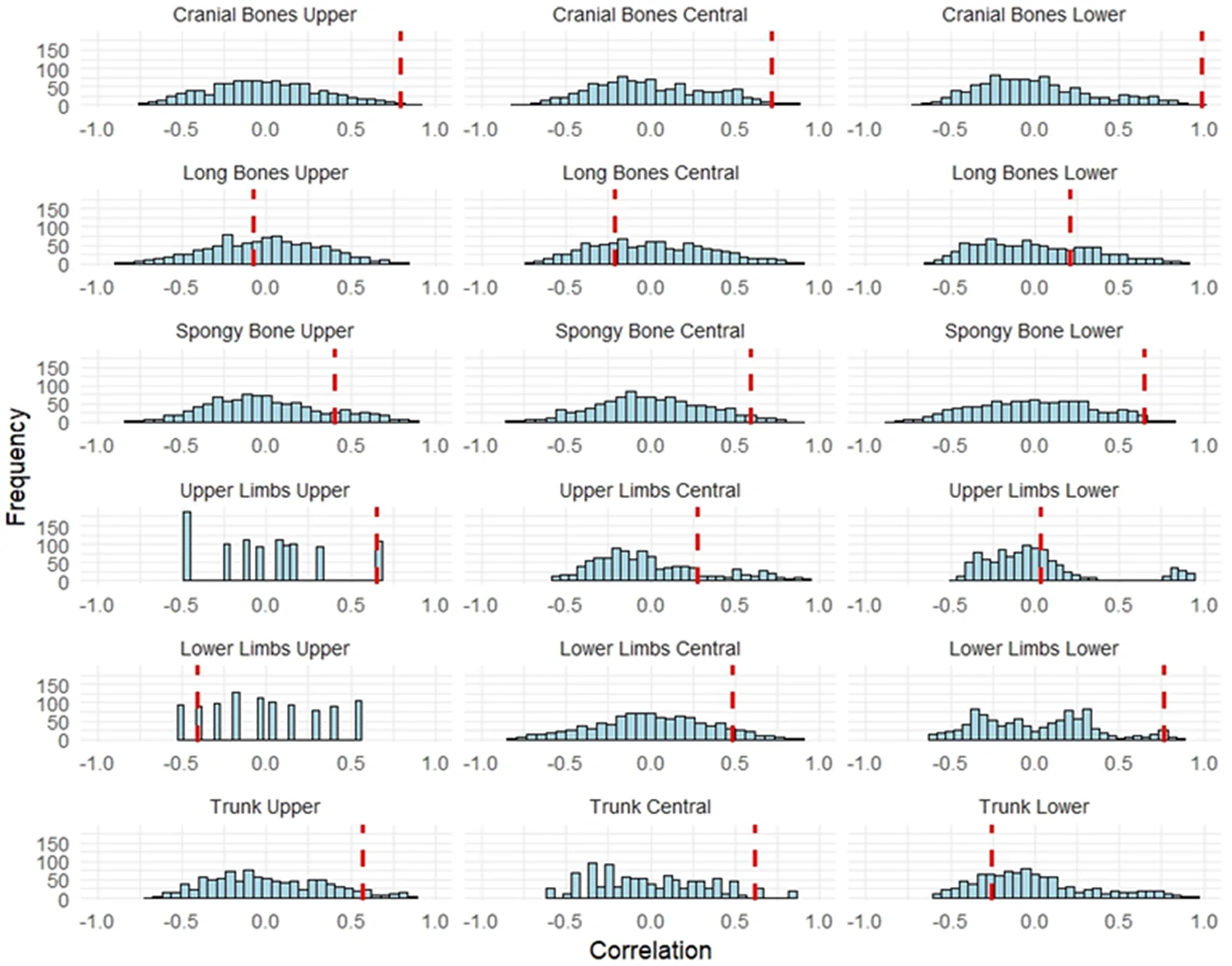
Plot of the permutation analysis correlations for vertical distribution. Each panel shows the distribution of correlations generated by randomly permuting the virtual dataset 1000 times. The red dashed line marks the observed correlation for each subgroup (calculated before permutation).

**Fig 14:**
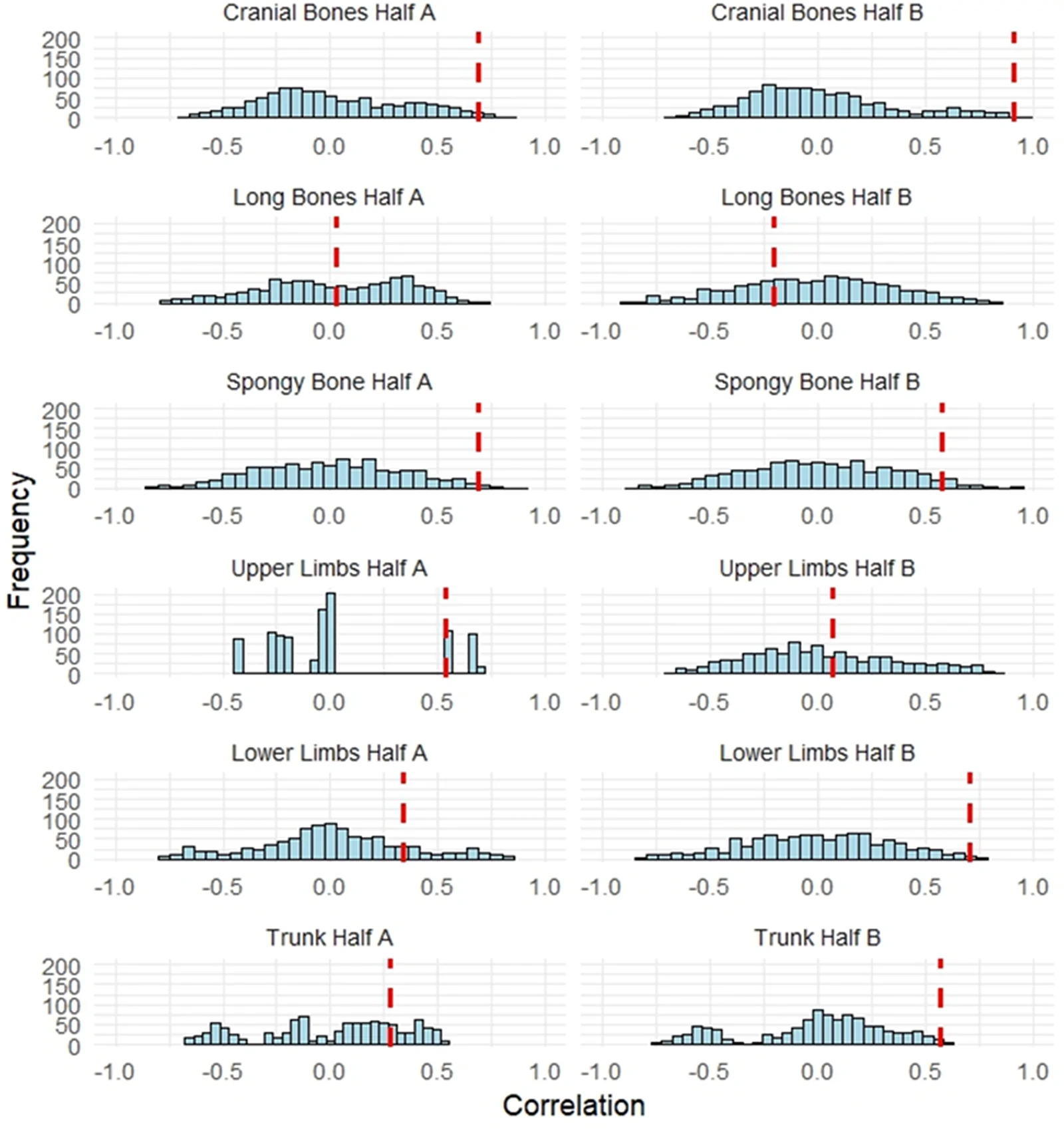
Plot of the permutation analysis correlations for horizontal distribution in half A and B. Each panel shows the distribution of correlations generated by randomly permuting the virtual dataset 1000 times. The red dashed line marks the observed correlation for each subgroup (calculated before permutation).

**Fig 15:**
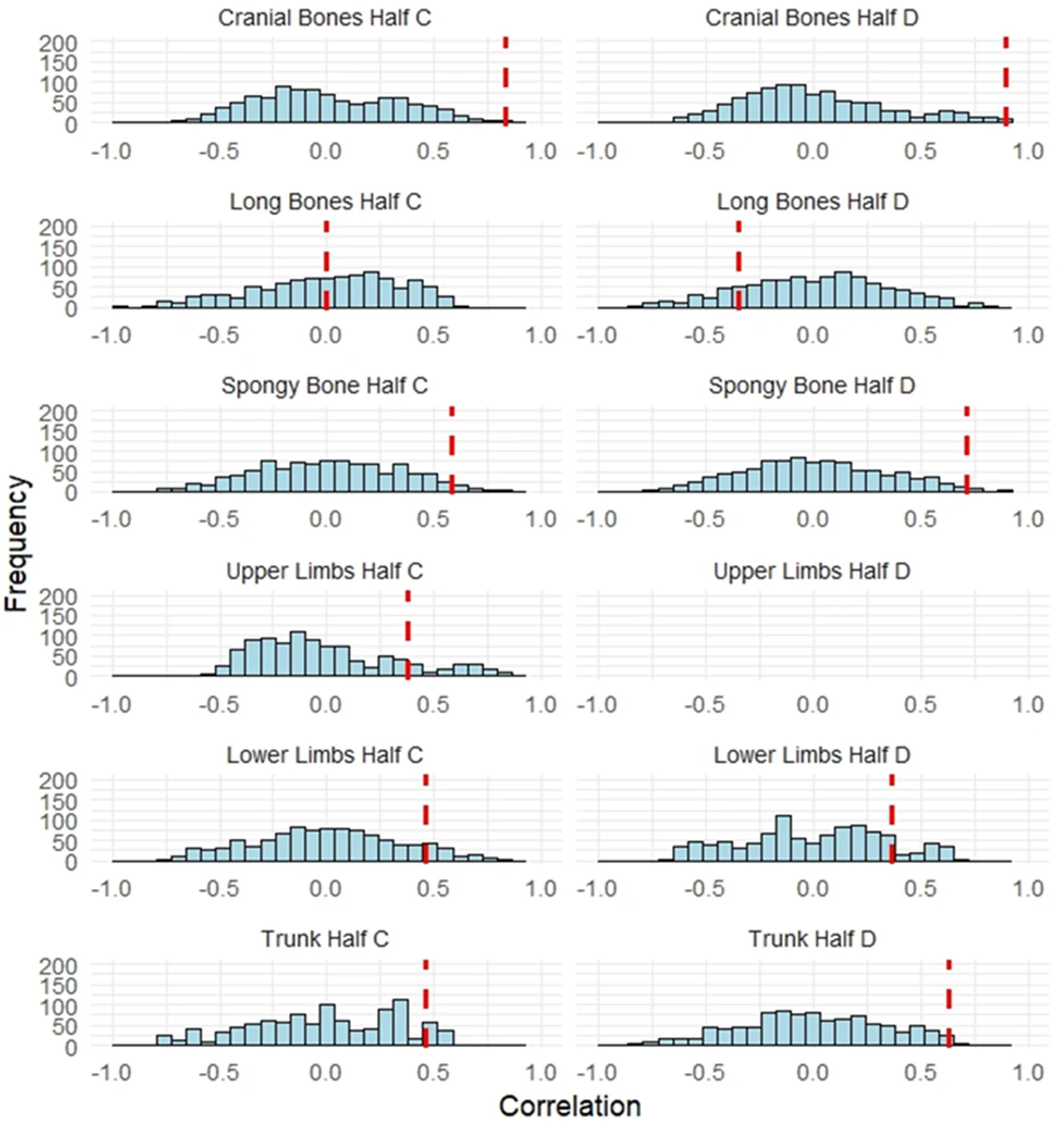
Plot of the permutation analysis correlations for horizontal distribution in half C and D. Each panel shows the distribution of correlations generated by randomly permuting the virtual dataset 1000 times. The red dashed line marks the observed correlation for each subgroup (calculated before permutation).

The plotting of the red dashed line in Fig 13, which represents the observed correlation for each subgroup, appears outside the bulk of the null distribution for all the cranial variables and for upper and lower limbs in the upper and lower sections, respectively. The same might be true for ‘Spongy Bone Lower’ and ‘Trunk Central’. Regarding the horizontal distribution, a similar pattern is observed for the cranial remains in all halves as well as for spongy bone in half A, lower limbs in half B, trunk remains in half B and D, and spongy bone in half D (Figs 14, 15). Even though the permutation analysis does not assume normality, it is worth observing that the simulated correlations show skewed distributions in limbs and trunk categories.

## 4. Discussion and conclusions

This study highlights the potential of Computerized Tomography (CT) as a tool for investigating cremation burials and aiding their excavation [4,9,10,13,16,55]. However, it also highlights that technical limitations hinder scanning complex objects. Despite systems designed for large items, dense materials present challenges. Higgins and colleagues [10] demonstrated that higher voltages (450 kV) are crucial for clear imaging of vessel contents. Unfortunately, advanced CT scanners are rare in universities due to cost and are primarily owned by private companies. Moreover, CT scans, though non-destructive [11], especially with little chance of preserved DNA [56], may pose risks when moving fragile artefacts. Transporting large archaeological samples to specialized centres is expensive and sometimes restricted by authorities.

All these factors can result in the need to use less sophisticated scanning machines, producing lower-quality images that hinder the testing of automatic segmentation tools. Beyond scanning limitations, this research demonstrates that image post-processing is equally of pivotal importance. While raw data quality may be constrained, corrections and filters can enhance image clarity, enabling qualitative and quantitative observations. Moreover, allocating more time for scanning can be advantageous, particularly with versatile systems, as demonstrated in the case of burial 15. Logistical and time constraints may limit such adjustments, but case-by-case evaluations could prove beneficial.

New approaches have been developed to enhance automatic segmentation through machine-learning models and clustering methods [6,57]. The recent versions of common imaging software, such as Avizo (Thermo Fisher Scientific) and ImageJ [34] incorporate these applications. While effective in contexts outside cremation urns, these approaches often rely on meticulously segmented input images and good-quality scans. Manual segmentation remains necessary for complex objects with poor-quality images where grayscale values overlap, making feature boundaries hard to define [58,59]. These processes are time-intensive, requiring detailed corrections and oversight. Many recent models were tested on different samples like fossils, mummies, organs, and hard non-biological materials [59,60] and were demonstrated to improve automatic segmentation. However, even though some advancements have been made specifically on cremation samples [6], a straightforward method that does not require a strong background in imaging is still lacking for this type of remains.

The presented protocol ensures efficient, controlled segmentation by processing five evenly spaced slices per view across the bone layer. While small elements like teeth or phalanges may be missed due to slice intervals (2–5 cm, typically ≤ 3.5–4 cm), most diagnostic elements, generally larger than 2 cm, are effectively segmented without requiring full quantitative analysis. The segmentation process required approximately 3–5 days per urn, depending on the quality of the tomographic images. Based on the authors’ experience, this time frame is generally feasible, although it is influenced by the observer’s familiarity with tomographic image interpretation. An initial learning period should be expected, and the first segmentation is therefore likely to require more time than subsequent ones. For this reason, the authors recommend starting with the highest-quality scans before progressing to more challenging datasets, allowing experience to be gradually acquired. When planning a project, the time required for segmentation should be carefully considered, particularly in relation to the number of urns to be analysed and the resources available. While the proposed workflow is feasible for studies of comparable scale, it may become time-consuming when applied to very large datasets. On the other hand, projects with sufficient funding to acquire a large number of CT scans are also more likely to have the resources necessary for the corresponding image post-processing.

The tomographic images proved to be valid diagnostic tools, consistent with similar studies. They revealed sediment dehydration, indicating clay-rich soil, and identified the position of remains, potential grave goods [4–6,9,10,13,14,16], and vessel conditions [4–6,15]. However, dense clay-rich soil absorbed X-rays, obscuring remains in some cases. Waltenberger and colleagues [16], who scanned two vessels from an Austrian site *en-bloc*, reported a similar difficulty*.* For burials 29 and 30, scans revealed fewer remains than physical analysis. In burial 30, collapsed ceramic fragments, probably fragments of a lid that collapsed into the vessel under the soil pressure, likely shielded some remains during the scanning process that were later identified during excavation.

In burial 29, the lack of evidence for skeletal remains in the CT scan likely stems from the vessel containing a young non-adult individual (3–5 years). Most remains were just millimetres thick and embedded in dense soil. This 20 cm high urn was taller than others for non-adults (11–15 cm). This observation becomes even more relevant considering that, despite the very young age of the individual, burial 29 was found at the centre of the smaller tumulus, in a prominent position (Fig 1). In contrast, the urn from burial 15, at the centre of the larger tumulus, was less preserved probably due to the collapse of its upper portion under sediment pressure. However, the collapsed walls of the vessel shielded the bones from further soil intrusion. The presence of compressed remains and sediment between bones in all other urns, except burial 26, indicates non-perishable lids were rarely used, consistent with findings of Cavazzuti and Salvadei [61].

Tomographic images effectively estimated the biological profile of individuals, generally predicting the MNI accurately. A second individual in burial 19 could not be confirmed from scans, as it was unclear if three petrous bone fragments were from the same bone. Post-excavation, these fragments confirmed at least two individuals, one non-adult (as hypothesized) and one adult. Age was correctly assessed in 7 of 10 cases: indeterminate results were linked to poor image quality (burials 29, 30, 32). Scans proved to be ineffective for sex estimation. However, given the strict methodological protocol applied (see paragraph 1.2.4) and poor preservation of diagnostic features, the sex of only one individual could be physically estimated.

Thermal alteration to bones, including transverse, longitudinal, and curved fractures [36] was predicted from scans, except in burial 29 due to poor image quality. Fractures were not quantified virtually, as counting them in each view (XY, XZ, YZ) on every element without 3D segmentation would be highly time-consuming. For general taphonomic assessment, the qualitative virtual approach proved reliable [10], with high fracture frequency confirmed in physical analysis. Concentric fractures and warping are less common, while mosaic fractures, generally prevalent on flat bones (e.g., cranium) [35,36], are harder to observe in low-quality tomographic images due to their fine cortical texture. The estimated combustion temperature could not be inferred solely from virtual analysis, but the presence of various fracture types and deformation suggests the process exceeded the dehydration phase (starting around 100 °C) and entered the decomposition phase (ending around 800 °C), where water loss and collagen destruction cause fractures [35,37,62]. Physical analysis confirmed most bones were calcined, consistent with burning temperatures of at least 700/800 °C [40].

As far as the authors are aware, this study presents the first detailed quantitative analysis of horizontal and vertical distribution of remains in vessels using both virtual and physical approaches. Virtual analysis accurately predicted cranial and long bone vertical and horizontal distribution, with discrepancies in five cases for the cranial bones and three cases for the long bones. Greater variation in long bone percentages arose as the physical analysis reclassified some bones into limb categories. Discrepancies in cranial bone predictions were linked to the poor-quality scans of burials 19, 29, and 30, and, only for the vertical distribution, to burial 15’s collapsed urn, which compressed the remains, obscuring them in tomographic images.

Spongy bone predictions were less accurate, with half of the results showing opposite vertical distributions to the physical analysis results. For the horizontal distribution, the prediction appeared slightly more precise, even though the prevalence of trabecular bone in half A and C for burial 30 was not confirmed by the physical quantification. This discrepancy likely stems from trabecular bone brittleness, leading to fragmentation during excavation [63] and handling [2], increasing counted elements. Moreover, some spongy elements could be reassigned to more specific anatomical categories post- excavation. Few limb and trunk remains were identified in scans, and predictions relied on higher-quality images (e.g., burials 8, 15, 16, 26), highlighting the importance of image quality. Permutation analysis generally supports these findings. Cranial bone distribution predictions showed a low likelihood of being random, and proved to be the most reliable, while long bone values exhibited low correlations between virtual and physical analyses, likely due to higher variation in predicted percentages. The prediction of spongy bone distribution is the second most consistent, with positive and significant correlations observed in two horizontal (half A and half D) and one vertical section (lower).

The analysis revealed moderate positive correlations values for upper and lower limbs, with significant p-values, though the results were less meaningful due to limited observations. For the trunk remains, consistent predictions were observed in two cases (burial 26 in half B and burial 8 in half D), but weaker correlations were noted compared to other categories. The distributions of upper and lower limbs appear skewed due to few elements counted, especially in the virtual method, affecting accuracy. If the datasets are small, the random shuffling likely does not fully sample all possible combinations. The virtual analysis was more effective in predicting horizontal distribution than vertical distribution, though the difference was minimal. Vertical space divided into three sections reduced accuracy, with a 33.3% probability of correct placement, compared to 50% for horizontal distribution. Quarters could have been calculated for the virtual horizontal distribution instead of halves. However, the high fragmentation of the data and the many spreadsheets produced and combined would have considerably increased data manipulation and made the process more prone to error.

**Fig 16:**
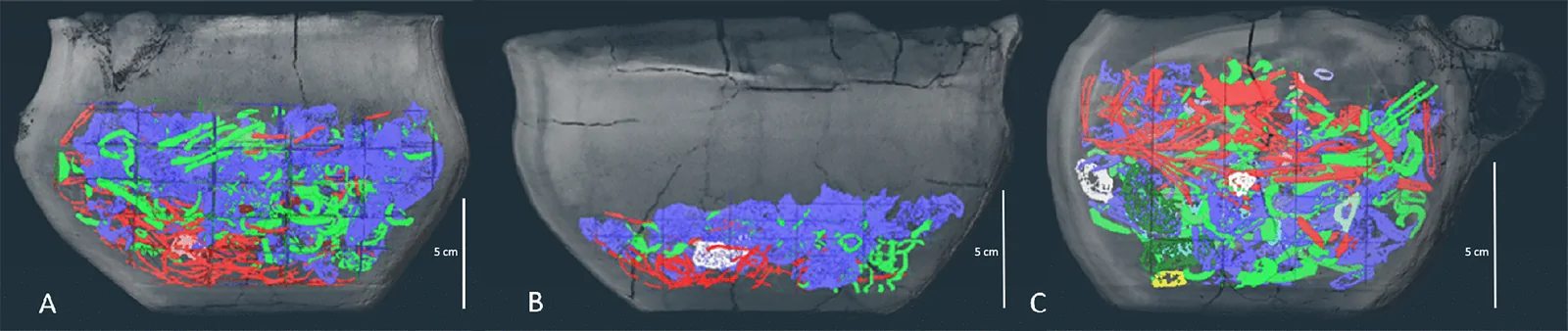
Burial 19 (A), 20 (B), 26(C). Red: cranial remains; green: long bones; blue: spongy bone. Cranial remains appear prevalent at the bottom of the vessel in burial 19 and 20, and at the top in burial 26.

Identifying intentional placement trends from numerical data alone is difficult, particularly when the prevalence of a class of remains in a specific area is moderate (e.g., ~ 60%). However, marked patterns (e.g., ≥ 70% of elements in one section) may suggest intentionality. For instance, in burial 15, despite even percentages, at least five vertebral bodies clustered on one side of the vessel. In this regard, the horizontal distribution analysis is limited by the inherently arbitrary division of the urns into halves. Although consistent virtual axes were used to ensure comparability across different vessels and between digital and physical observations, there is no certainty that these divisions correspond to any intentional organisation of the remains. This issue was anticipated, but without identifiable reference points on the vessels, avoiding subdivision entirely would have increased the risk of overlooking possible evidence of deliberate placement. For this reason, the method was maintained, with the results interpreted as suggestive rather than conclusive. Conversely, this visual prevalence was consistent with the quantitative data for the cranial vertical distribution in the cases of burials 19, 20, and 26 (Fig 16), demonstrating the virtual approach’s ability to integrate qualitative and quantitative findings.

While some disturbance of the remains during excavation, transport, or handling cannot be entirely ruled out, substantial displacement is unlikely. The surrounding soil was extremely compact, and even if it had been less so at the time of excavation, its density and weight would still have limited any internal movement. Furthermore, the urns were well preserved and carefully secured during recovery, making it improbable that their contents shifted substantially afterward. The lack of any visible changes between the scanning and the subsequent excavation supports this assessment.

The approach outlined here is not intended to resolve inherent challenges of working with cremated remains, such as their fragmentary nature, or the limitations of tomographic imaging, including low resolution or overlapping material densities. Instead, its purpose is to offer a practical method for navigating these constraints while still extracting meaningful qualitative and quantitative information. Because the procedure was developed and tested on medium- and low-quality scans and relies on a relatively simple segmentation workflow, it can be applied in a wide range of situations. This is especially relevant in contexts where access to facilities, funding, secure long-distance transport, or specialists with extensive virtual analysis expertise is restricted.

The choice of analytical approach should always take into account the available time and financial resources, as these may constrain the methods that can realistically be applied. When sufficient resources are not available to ensure an adequate investigation, leaving urns unexcavated until proper analysis can be carried out may be the preferable option. Conversely, when resources permit, the integration of CT scanning can substantially improve the investigation, particularly when at least medium-quality images are available. As demonstrated in the present study, even in the absence of a complete quantitative analysis, tomographic images can provide valuable qualitative information on the biological profile of the individuals and aspects of funerary behaviour. They can also enhance documentation, guide the excavation strategy, and support the careful planning of extraction units (e.g., layers and quadrants), thereby allowing detailed information on the spatial distribution of the remains within the urns to be recorded while minimising further fragmentation and damage during recovery.

Given the variability typical of archaeological contexts, including differences in preservation conditions, excavation practices, soil characteristics, and the size and shape of urns, it is impossible to anticipate all circumstances in advance. Nonetheless, although this method was developed using one group of urns from a single archaeological site, it should be transferrable to other sites as long as tomographic scanning is available, the vessels can physically be accommodated in the machines, and the resulting images are sufficiently interpretable. The number of slices chosen for segmentation may differ depending on vessel size, the configuration of the bone deposit, and the amount of analysis time available.

Despite the limitations discussed above, this study offers the most comprehensive dataset on urned cremations analysed via tomographic images. Building upon previous methodological applications, such as that of Higgins et al. [10], who provided a detailed analysis on a single case, the present study demonstrates the potential of this approach across multiple urns, highlighting the variability of preservation conditions, analytical challenges, and information that can be obtained from different burials. Although virtual data can provide insights into taphonomy, thermal alteration, biological profiles, and remains distribution, physical analysis remains essential, especially when high-quality tomographic images are unavailable [5,10,16]. However, scans can guide excavations, preventing bone damage and simplifying the process, making this approach valuable in contexts where excavation is restricted due to ethical or preservation concerns [64].The evolution of virtual analysis for cremated remains will rely on machine-learning approaches designed for segmentation and analysis, even on medium-quality images. Higher-quality scans would enable further research on assessing thermal alterations to bones.

## Supporting information

S1 Table List of urns selected for the scanning, including sizes and shapes. (XLSX)

S2 File Required ‘Inclusivity in global research’ questionnaire. (DOCX)
